# Evaluation of serum and dietary profiles of vitamin B12 and folate and their association with systemic complications in patients with Crohn’s disease

**DOI:** 10.1038/s41430-026-01724-3

**Published:** 2026-03-27

**Authors:** Marina Moreira de Castro, Vitor Nascimento dos Santos, Maysa Santos Gomes, Aline Dias Gonçalves Ferraz, Leticia Martins Ignacio-Souza, Marcio Alberto Torsoni, Adriana Souza Torsoni, Maria de Lourdes Setsuko Ayrizono, Raquel Franco Leal, Ligiana Pires Corona, Marciane Milanski

**Affiliations:** 1 Laboratory of Metabolic Disorders, School of Applied Sciences, University of Campinas (Unicamp), Limeira, São Paulo Brazil; 2 Inflammatory Bowel Disease Research Laboratory, Gastrocenter, Colorectal Surgery Unit, School of Medical Sciences, University of Campinas (Unicamp), Campinas, São Paulo Brazil; 3 Laboratory of Nutritional Epidemiology, School of Applied Sciences, University of Campinas (Unicamp), Limeira, São Paulo Brazil

**Keywords:** Crohn's disease, Nutrition

## Abstract

**Background/objective:**

Crohn’s Disease (CD) is a chronic inflammatory bowel disease that affects micronutrient levels, impacting metabolic pathways. Homocysteine (Hcy) levels, regulated by folate and vitamin B12, are associated with cardiovascular and extraintestinal manifestations (EIMs). This study aims to analyze the dietary intake and serum concentrations of folate and vitamin B12 and their relationship with systemic complications in patients with CD in remission and active phases.

**Methods:**

This cross-sectional study included 60 patients, and nutrient intake was stratified into tertiles by disease phase. The metabolic safety of folate and B12 was evaluated by characterizing metabolic risk patterns associated with elevated Hcy. Statistical analyses evaluated associations between dietary intake, metabolic safety, and disease phenotype.

**Results:**

Vitamin B12 intake was negatively associated with erythrocyte sedimentation rate and positively associated with the absence of EIMs. Serum folate and non-HDL cholesterol levels were lower in the active group. Clinical vitamin B12 deficiency was more frequent in active disease, contributing to an elevated Hcy risk. Stricturing/penetrating phenotypes showed lower vitamin B12 levels compared to non-stricturing, non-penetrating phenotypes. Additionally, 96% of patients with structuring/penetrating disease were at high Hcy risk, while 30% of patients with non-stricturing, non-penetrating disease were in the lowest risk category.

**Conclusions:**

The data suggest that folate and B12 levels could be markers for clinical characteristics and associated metabolic risk in patients with CD. Our study highlighted associations that may justify the importance of nutritional follow-up and biochemical assessments in the clinical routine of patients with CD.

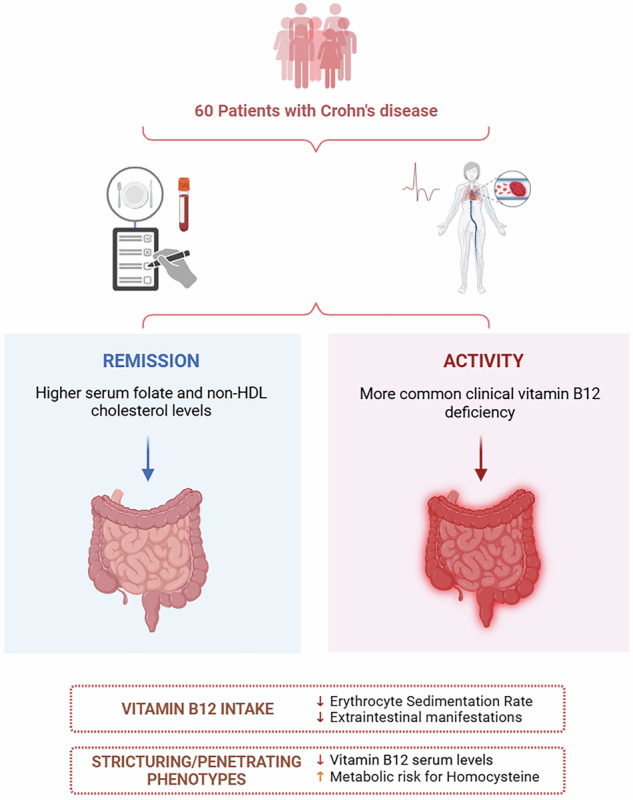

## Key points

- Patients with stricturing/penetrating disease had significantly lower vitamin B12 levels and a higher risk of elevated homocysteine compared to those with non-stricturing, non-penetrating disease.
- Active Crohn’s disease is linked to lower serum folate and non-HDL cholesterol levels, as well as higher prevalence of clinical vitamin B12 deficiency, contributing to an increased metabolic risk of elevated homocysteine.
- Folate and vitamin B12 levels are potential markers for clinical monitoring and the management of metabolic risks in Crohn’s disease.

## Introduction

Crohn’s disease (CD) is a chronic inflammatory bowel disease (IBD) characterized by transmural inflammation that can affect any part of the gastrointestinal tract in a discontinuous manner. This can result in complications such as strictures, fistulas, and abscesses [1]. Although the cause of IBD is still unclear, the multifactorial etiology of CD includes genetic, environmental, and immunological factors, as well as the gut microbiota [2]. Moreover, IBD is associated with extraintestinal manifestations (EIMs) - such as rheumatological, dermatological, or ophthalmological manifestations [3].

Nutritional status is essential in the management of CD. Many patients can experience malabsorption, weight loss, protein-losing enteropathy, or increased energy demand. While malnutrition has always been associated with CD, recent evidence has shown the growing prevalence of overweight and deficiencies in specific micronutrients, which may be related to drug interactions, inadequate intake, or impaired absorption due to inflammatory processes [4, 5].

Since the distal ileum is the part commonly affected by the disease, which is also the primary site for vitamin B12 absorption, vitamin B12 deficiency and malabsorption are frequently observed in patients with CD [5, 6]. Folate (vitamin B9) and vitamin B12 are essential nutrients in many metabolic processes, such as nucleotide synthesis, biological methylation maintenance of erythropoiesis, nervous system functions, and homocysteine (Hcy) metabolism. Deficiencies in these vitamins may lead to macrocytic anemia, neurological disorders, and hyperhomocysteinemia [7–9]. Furthermore, elevated Hcy due to folate and vitamin B12 deficiency has been associated with increased cardiovascular disease risk and thromboembolic events in CD [10].

Considering the risks of insufficient dietary intake of folate and vitamin B12, and the limited understanding of the relationship between these nutrients and systemic complications in CD, our aim was to analyze the serum and dietary profile of folate and vitamin B12 and their correlation with systemic complications, including metabolic risk characterizations of Hcy in relation to cardiovascular parameters and EIMs in patients with CD. Exploring the correlations between nutrients, cardiovascular risk, and EIMs is essential for managing CD, since nutrient depletion may increase intestinal and metabolic risks contributing to CD complications.

## Materials and methods

### Study population

This cross-sectional study included patients with CD aged between 18 and 60 years, recruited at the IBD outpatient clinic of the Colorectal Surgery Unit at the Gastrocenter, University of Campinas (Unicamp), between May 2017 and July 2018. The sample size was estimated based on an expected difference of at least 2 body mass index (BMI) points between the remission and activity groups, with a maximum type I error of 5% and minimum statistical power of 80% [11]. This indicated a minimum of 16 participants per group; to account for possible exclusions or incomplete data, we aimed to enroll at least 30 patients per group.

To be eligible for the study, patients needed to have a confirmed diagnosis of CD and to have undergone endoscopic or imaging evaluations within two months before recruitment, after which they were invited to participate. The diagnosis of CD was confirmed through a combination of endoscopic, radiological, and histological criteria. Exclusion criteria included women who were pregnant or breastfeeding, patients with edema, and those without recent endoscopic or imaging evaluations. All patients provided their written informed consent before the interview and data collection. The Ethics Committee of the University of Campinas (Unicamp) approved this study (CAAE n°62802016.0.0000.5404).

### Disease activity, classification, clinical and demographic data

The phase of CD was assessed either through colonoscopy using the Crohn’s Disease Endoscopic Index of Severity (CDEIS) [12], defined as an activity with a score of ≥5 or the presence of deep ulcers in at least one intestinal segment, or by magnetic resonance enterography, which identified deep ulcers, edema, and creeping mesenteric fat near the affected intestinal area. Then, patients were grouped into remission and activity. The disease location and behavior were assessed using the Montreal classification [13]. Clinical and demographic data were obtained based on a participant interview and a review of medical records, as detailed in previously published studies [14, 15].

### Dietary and biochemical parameters

A 24-hour dietary recall was applied to obtain patient dietary intake information about all foods and beverages consumed the day before the interview. Household measures were used during the interview to help patients visualize portion sizes. According to Brazilian food composition tables, all portion sizes were converted into grams and milliliters [16–18]. Subsequently, the dietary intake data were analyzed using the Nutrition Data System for Research software (version 2019), developed by the Nutrition Coordinating Center (NCC), University of Minnesota, Minneapolis, MN. Nutrient intakes were then estimated and stratified into tertiles according to disease phase.

To assess the prevalence of inadequate nutrient intake, the adequacy of folate and vitamin B12 was calculated using the Dietary Reference Intakes (DRI) as reference values, specifically the Estimated Average Requirement (EAR): 2 µg for vitamin B12 and 320 µg for folate in adults ≥19 years and 330 µg for those ≤18 years [19, 20]. Energy and protein intake adequacy was evaluated according to the European Society for Clinical Nutrition and Metabolism (ESPEN) guidelines. Energy requirements were defined as 30–35 kcal/kg/day, and protein requirements were defined by disease activity: 1 g/kg/day during remission and 1.2–1.5 g/kg/day during activity [21].

The biochemical data evaluated included serum folate, vitamin B12, total cholesterol, and non-HDL cholesterol (non-HDL-C). Serum folate, vitamin B12, and total cholesterol were obtained during routine patient follow-up or from medical records and were included only when performed within two months before or after the nutritional assessment. Based on total cholesterol values, non-HDL-C levels were calculated following the reference values from the dyslipidemia and atherosclerosis prevention guideline of the Brazilian Society of Cardiology [22]. These reference values were applied to verify and assess the adequacy of these components, as well as ensure their clinical relevance in the analysis.

### Evaluation of the metabolic marker of micronutrients: homocysteine

Homocysteine, a marker of metabolic health, was indirectly evaluated through the serum levels of its regulatory micronutrients – vitamins B12 and B9 – consistent with the evaluations used in previous studies [23–25]. For this purpose, clinical studies, large cohort studies, and a validated population-based study using data from the National Health and Nutrition Examination Survey (NHANES) were used to establish the reference range for folate and vitamin B12 [23–25].

Based on this, the following categories for B9 levels based on Hcy were established: clinical deficiency (<3 ng/mL), risk of insufficiency (3–4 ng/mL), metabolic safety (> 4 ng/mL), and elevated (>20 ng/mL). Regarding vitamin B12, the following categories were used: clinical deficiency (<140 pmol/L), metabolic deficiency with elevated Hcy (140–300 pmol/L), metabolic risk (300–400 pmol/L), and metabolic safety (>400 pmol/L).

### Statistical analysis

The descriptive analysis of the variables was performed using the relative frequency distributions, mean, and standard deviation for continuous variables, and proportions were estimated for categorical variables. The Shapiro–Wilk test was applied to assess the normality of the variables. For data that followed a normal distribution, a parametric test like Student’s *t* test was used; otherwise, a non-parametric test such as the Mann–Whitney test was applied. Comparisons were performed within each tertile between the remission and activity groups. Categorical variables were compared using the Chi-Square (χ²) test or Fisher’s exact test, as appropriate. Due to the small number of patients in the penetrating (B3) category, B2 and B3 behaviors were combined for statistical purposes to ensure adequate group size and analytical stability.

Multinomial logistic regression analysis was performed to explore the association between nutrients with clinical and demographic variables. The significance level of *p* < 0.05 was applied for all analyses, and data were analyzed using Stata® software, version 14.

## Results

### Characteristics of the patients

Our database includes 60 patients with CD, with 29 in the activity group and 31 in remission evaluated using the CDEIS score. In the activity group, 51.61% of the patients were male, with a mean age of 33.82 years. In the remission group, 55.17% of the patients were female, and the mean age was 39.67 years.

According to the Montreal Classification, most patients had the non-stricturing, non-penetrating phenotype (B1): 48.39% in remission and 55.17% in the activity group. The stricturing phenotype (B2) was observed in 41.94% of patients in remission and 31.03% in activity, and the penetrating phenotype (B3) was observed in 9.68% of patients in remission and 13.79% in activity. The demographic and clinical characteristics of the patients can be found in our previous study [14].

### Assessment of dietary intake

Initially, we compared dietary intake across tertiles between those in remission and those with active disease, as presented in Table 1. There was no significant difference in macronutrient intake (carbohydrate, fat, and protein) between the groups, despite carbohydrate consumption being below the recommended range (45–65% of energy) [20] in T1 in both groups.

**Table 1:** Analysis of dietary intake across tertiles between the remission and activity groups, presented as means (standard deviation).

Nutrients	Remission			Activity					
	T1	T2	T3	T1	T2	T3	*p-*value T1	*p-*value T2	*p-*value T3
Energy (kcal)	1446 ± 203	2066 ± 158	2923 ± 349	1276 ± 344	1968 ± 195	2899 ± 610	0.177	0.232	0.922
Fat (% of energy)	26.5 ± 2.8	34 ± 1.6	42.1 ± 2.9	26.2 ± 3.6	34 ± 1.7	42.4 ± 4.2	0.837	0.946	0.842
Carbohydrate (% of energy)	35.7 ± 4.3	46.5 ± 2.7	56.5 ± 3.2	37.2 ± 3.4	46.2 ± 3.5	56.7 ± 4.5	0.406	0.819	0.943
Carbohydrate (g/d)	155.4 ± 25	226.9 ± 26.6	316.2 ± 45.1	154.4 ± 41.6	236.9 ± 25.9	369.5 ± 88.8	0.790	0.226	0.086
Protein (% of energy)	13.3 ± 3	19.3 ± 1.6	24.9 ± 2.7	14.7 ± 1.6	19.6 ± 1.1	24.6 ± 2.5	0.231	0.639	0.810
Protein (g/d)	55 ± 16.3	98.6 ± 10.9	142.3 ± 21.4	60.1 ± 17.7	94.4 ± 10.7	149.9 ± 28.7	0.516	0.398	0.520
Animal Protein (g/d)	32.5 ± 12.7	62.7 ± 11	111.2 ± 19.9	37.2 ± 9.7	65.6 ± 8.8	123.8 ± 27.4	0.305	0.470	0.283
Vegetable Protein (g/d)	18.6 ± 3.7	26.8 ± 3	44.7 ± 6.8	14.8 ± 5.7	25.4 ± 2.2	38.9 ± 5.4	0.089	0.238	0.056
Vitamin B12 (µg)	1.8 ± 0.6	3.5 ± 0.8	7.2 ± 2.5	1.9 ± 0.4	3.8 ± 1	16.6 ± 24.2	0.909	0.568	0.098
Total Folate (µg)	223.7 ± 42.5	357.8 ± 44.8	537 ± 66.9	224.5 ± 77.4	367.5 ± 53.5	557.3 ± 100.3	0.976	0.667	0.607
Dietary Folate Equivalents (µg)	293.9 ± 60.4	480.5 ± 59	740.4 ± 130.7	275.4 ± 96.8	477.7 ± 72.8	757.6 ± 181.3	0.602	0.927	0.814
Natural Folate (food folate) (µg)	115.2 ± 33.5	184.2 ± 21.2	287.5 ± 52.8	99.6 ± 30.8	191.7 ± 21.7	301.5 ± 74.5	0.164	0.470	0.744
Synthetic Folate (folic acid) (µg)	86.1 ± 36.8	154.5 ± 27.4	306.7 ± 95.1	76.7 ± 32	157.2 ± 32.9	305.2 ± 117.7	0.354	0.909	0.624

Comparisons were performed within each tertile between the remission and activity groups.

Student’s *t* test performed for parametric variables: energy, fat, carbohydrate (% of energy), protein (% of energy), protein (g/d), vegetable protein, total folate, dietary folate equivalents. Mann-Whitney test performed for non-parametric variables: carbohydrate (g/d), animal protein, vitamin B12, natural folate, synthetic folate.

*T1* tertile 1, *T2* tertile 2, *T3* tertile 3, *SFA* saturated fatty acid, *MUFA* monounsaturated fatty acid, *PUFA* polyunsaturated fatty acid.

Moreover, we did not find differences in the consumption of vitamin B12 and folate when comparing patients at both phases of the disease.

Regarding protein intake among the patients, the dietary profile is characterized by high protein consumption, regardless of the disease phase. According to the ESPEN guidelines [21], a high protein intake was observed in 67.74% of patients in remission and 39.29% in active disease. Despite this, 22.58% of patients in remission and 32.14% in activity had protein intake below the recommendation, and only a smaller proportion of patients had adequate protein intake (Table 2).

**Table 2:** Level of adequacy of selected nutrients intake.

Nutrients	Remission		Activity		*p*-value
	Adequate (%)	Inadequate (%)	Adequate (%)	Inadequate (%)	
Energy (kcal/kg/day)	19.35	58.06 (below)	10.34	48.28 (below)	0.288
		22.58 (above)		41.38 (above)	
Protein (g/kg/day)	9.68	22.58 (below)	28.57	32.14 (below)	0.061
		67.74 (above)		39.29 (above)	
Vitamin B12 (µg)	87.10	12.90	82.14	17.86	0.597
Folate (µg)	100	0	100	0	N/A^a^

Energy requirements according to the ESPEN guidelines [21]: 30–35 kcal/kg/day.

Protein requirements according to the ESPEN guidelines [21]: Remission = 1 g/kg/day; Activity = 1.2–1.5 g/kg/day.

Dietary Reference Intakes (DRI) [19]: Adequate intake for vitamin B12 (Estimated Average Requirements: 2 µg); adequate intake for folate (Estimated Average Requirements: 320 µg for adults ≥19 years, 330 µg for those ≤18 years).

^a^*p*-value was not calculated because all patients were in the same category (100% in the same adequacy group).

Among patients in remission, only 19.35% achieved the recommended energy intake, while the majority (58.06%) consumed less, according to ESPEN recommendations [21]. In the active disease group, energy adequacy was even less frequent, with only 10.34% of patients meeting the recommendation, while 48.28% of them had energy intake below and 41.38% above the recommended levels.

According to DRI recommendations [19], adequacy intake for vitamin B12 was observed in 87.10% of patients in remission and 82.14% of those in active disease; and all patients in both groups met the adequacy requirements for folate.

Table 3 presents the associations between dietary intake and clinical and biochemical variables according to nutrient intake tertiles. The intermediate tertile of vitamin B12 intake (T2) was negatively associated with erythrocyte sedimentation rate (ESR) and glycemic load. Additionally, vitamin B12 intake was positively associated with the absence of EIMs. In the highest tertile (T3), vitamin B12 intake also correlated positively with folate equivalent and riboflavin intake, indicating that patients with higher vitamin B12 intake tended to have fewer EIMs and greater consumption of these related micronutrients.

**Table 3:** Multinominal logistic regression analysis of the association between nutrient consumption and clinical and laboratory parameters in patients with Crohn’s disease.

Variables	β	IC 95%	*p*-value
Protein^a^			
Ileal location (T3)	4.146	(0.395; 7.898)	0.030
Symptoms 1 to 2 (T3)	4.213	(0.038; 8.387)	0.048
Calf circumference (T2)	0.418	(0.026; 0.81)	0.036
Pyridoxine (T2)	4.29	(1.368; 7.211)	0.004
Pyridoxine (T3)	9.396	(4.698; 14.095)	<0.001
Vitamin B12^a^			
Ileocolonic location (T3)	3.986	(0.337; 7.635)	0.032
No EIMs (T3)	5.156	(0.642; 9.67)	0.025
Erythrocyte sedimentation rate (T2)	−0.049	(−0.098; −0)	0.048
Riboflavin (T3)	22.169	(6.073; 38.264)	0.007
Total folate (T3)	−0.238	(−0.423; −0.052)	0.012
Folate equivalent (T3)	0.132	(0.029; 0.235)	0.011
Glycemic load (T2)	−0.065	(−0.129; −0.001)	0.045
Total Folate^a^			
Red/poultry meat (T2)	1.284	(0; 2.567)	0.050
Fruits (T2)	0.521	(0.068; 0.973)	0.024
No EIMs (T2)	−6.299	(−12.61; 0.012)	0.050
Vitamin D (T3)	−1.997	(−3.791; −0.204)	0.029
Vitamin B12 (T2)	−1.055	(−1.977; −0.134)	0.025
Vitamin B12 (T3)	−1.377	(−2.367; −0.387)	0.006
Riboflavin (T2)	19.775	(2.468; 37.083)	0.025
Riboflavin (T3)	27.691	(8.784; 46.598)	0.004
Iron (T2)	0.859	(0.139; 1.579)	0.019
Iron (T3)	1.571	(0.605; 2.537)	0.001
Methionine (T2)	−4.941	(−9.397; −0.484)	0.030
Methionine (T3)	−7.742	(−12.918; −2.566)	0.003

^a^Dependent variables (protein, vitamin B12, folate) were categorized into tertiles for analysis, using tertile 1 as the reference category.

*T2* tertile 2, *T3* tertile 3, *EIMs* extraintestinal manifestations.

Total folate was positively associated with the consumption of red/poultry meat, fruits, and dietary iron in T2, suggesting that these dietary sources may contribute to the intermediate folate levels observed in this study. Total folate also showed positive associations with dietary iron and riboflavin in T3 and a negative association with the absence of EIMs in T2.

### Analysis of laboratory parameters

Regarding the metabolic risk for elevated Hcy, there was a difference in the clinical deficiency of vitamin B12 between patients in remission and in active disease. Patients in the active group had a higher prevalence of clinical deficiency (<140 pmol/L), with 25.93% affected, compared to only 4.17% in the remission group (*p* = 0.033). The most frequent category for both groups was metabolic deficiency/metabolic risk (140–400 pmol/L).

A significant difference in mean serum levels of folate and non-HDL-C was observed between the remission and activity groups (*p* = 0.012; *p* = 0.003, respectively). The most common classification for folate levels in both groups was metabolic safety/elevated (> 4 ng/mL). Non-HDL-C fractions were included in the analysis to enhance understanding of its role in cardiovascular risk. According to the Brazilian Society of Cardiology classification for non-HDL-C, all patients in the active group were classified in the optimal category, whereas only 46% of patients in remission achieved this classification. Notably, a significant difference was observed between the remission and active disease groups when stratified by cardiovascular risk (Table 4).

**Table 4:** Comparison of biochemical parameters according to Crohn’s disease activity in patients in remission and active disease.

Variables	Remission	Activity	*p*-value
Vitamin B12 (pmol/L) (mean ± SD)	306.37 ± 155.63	300.09 ± 188.44	0.719
Clinical deficiency (%)	4.17	25.93	0.033
Metabolic deficiency/metabolic risk (%)	79.17	55.56	0.074
Metabolic safety (%)	16.67	18.52	0.863
Folate (ng/mL) (mean ± SD)	12.7 ± 4.09	9.82 ± 3.8	0.012
Clinical deficiency/risk of insufficiency (%)	0	11.11	0.092
Metabolic safety/elevated (%)	100	88.89	0.092
Non-HDL cholesterol (mean ± SD)	132.23 ± 37.03	92.92 ± 24.71	0.003
Very high/high (%)	15.38	0	0.127
Desirable (%)	38.46	0	0.010
Optimal (%)	46.15	100	0.001

Vitamin B12 categories were grouped as follows: Clinical deficiency (<140 pmol/L), Metabolic deficiency/metabolic risk (140–400 pmol/L), and Metabolic safety (>400 pmol/L). Folate categories were grouped as follows: Clinical deficiency/risk of insufficiency (<4 ng/mL) and Metabolic safety/elevated (>4 ng/mL).

Moreover, in Table 5, the nutrients were evaluated in relation to metabolic risk patterns, considering disease behavior according to the Montreal Classification. The mean vitamin B12 level in the non-stricturing, non-penetrating phenotype (classified as B1) was 363.88 pmol/L, while in the stricturing and/or penetrating phenotypes (classified as B2 + B3), the mean was 239.78 pmol/L. A significant difference was observed between the groups regarding vitamin B12 levels.

**Table 5:** Comparison of biochemical parameters according to the behavior of Crohn’s disease by Montreal classification.

Variables	B1	B2 + B3	*p*-value
Vitamin B12 (pmol/L) (mean ± SD)	363.88 ± 192.84	239.78 ± 121.61	0.004
Clinical deficiency (%)	11.54	20	0.406
Metabolic deficiency/risk of insufficiency (%)	57.69	76	0.166
Metabolic safety (%)	30.77	4	0.012
Folate (ng/mL) (mean ± SD)	11.33 ± 4.48	11.04 ± 3.94	0.806
Clinical deficiency/risk of insufficiency (%)	3.7	8.33	0.483
Metabolic safety/elevated (%)	96.30	91.67	0.483
Non-HDL cholesterol (mean ± SD)	124.15 ± 39.75	100.42 ± 300.44	0.092
Very high/high (%)	0	15.38	0.127
Desirable (%)	14.29	23.08	0.557
Optimal (%)	85.71	61.54	0.557

Vitamin B12 categories were grouped as follows: Clinical deficiency (<140 pmol/L), Metabolic deficiency/metabolic risk (140–400 pmol/L), and Metabolic safety (>400 pmol/L). Folate categories were grouped as follows: Clinical deficiency/risk of insufficiency (<4 ng/mL) and Metabolic safety/elevated (>4 ng/mL). B1: non-stricturing, non-penetrating behavior; B2 + B3: stricturing and/or penetrating behavior.

When stratifying the groups into Hcy risk pattern categories, a significant difference was also observed between the groups in the metabolic safety category, in which 30% of patients in the B1 group were in this category compared to only 4% of patients in the B2 + B3 group. In addition, most patients with stricturing and/or penetrating phenotype (B2 + B3) were classified in the higher metabolic risk categories for Hcy, with 20% in the clinical deficiency category and 76% in the metabolic deficiency and risk of insufficiency category.

## Discussion

Our study highlights the complexity of nutrient deficiencies and their associations with metabolic and clinical complications in patients with CD. The findings emphasize the importance of folate and vitamin B12 in managing systemic complications, including cardiovascular risks and EIMs.

Previous studies have primarily examined B12 and folate deficiencies in relation to clinical activity or anatomical factors. Akbulut reported lower B12 levels with ileal involvement [26], and Huang et al. identified ileal resection as a risk factor for B12 deficiency and shorter disease duration for folate deficiency [27]. Gioxari et al. evaluated serum vitamin status and inflammatory/oxidative biomarkers, although dietary intake was analyzed only as a determinant of vitamin levels [28]. In contrast, our study integrates dietary intake, serum concentrations, systemic complications, and disease behavior according to the Montreal classification, using objective endoscopic or imaging criteria to define disease activity. This approach allowed us to identify associations between vitamin B12 and folate intake, inflammation, EIMs, metabolic risk, and stricturing/penetrating phenotypes, providing a more comprehensive assessment of nutritional and clinical determinants in CD.

We demonstrated that patients had a high protein intake. Patients with active disease require increased protein due to intestinal losses, reduction in lean body mass, drug interactions, and inflammatory processes [21]. However, 67.74% of patients in remission also consumed a high-protein diet, which is relevant since excessive protein intake, particularly from animal sources, may have a deleterious effect on the gut microbiota, an essential component for the development and management of IBD [29]. An excess of nitrogenous compounds in the intestinal lumen stimulates the microbiota to produce harmful substances and promotes the growth of pathogenic strains [30]. Additionally, Lylowska-Szuber et al. have shown that dysbiosis significantly impacts cardiovascular risk in IBD through the production of inflammatory byproducts, increased intestinal permeability, and the promotion of low-grade inflammation [31].

High protein intake may not prevent muscle loss when caloric intake is low, contributing to sarcopenic obesity [21]. In this study, carbohydrate consumption in the lowest tertile (T1) was below recommendations, and diets rich in protein but low in carbohydrates may not provide adequate energy, especially when total energy intake is insufficient. A systematic review found that patients with CD typically consume less energy than recommended while meeting or exceeding protein requirements [32]. This imbalance can lead the body to use protein as an energy source, limiting its availability for muscle maintenance and impairing protein metabolism, particularly in chronic or hypercatabolic conditions [33]. Another relevant aspect is that as protein consumption increases, the substrate is available to form Hcy [34]. Since vitamins B12 and folate are essential for Hcy metabolism, deficiencies in these nutrients can raise Hcy levels, which are associated with increased cardiovascular risk [5, 35].

Although most patients had adequate intake of vitamin B12 and folate, a large proportion of them were found to be at risk for elevated Hcy levels based on their serum B12 levels. It is important to note that adequacy was assessed using Dietary Reference Intakes (DRIs) for healthy individuals [19], as we do not yet have specific reference values for IBD. This is concerning as patients with CD often have increased nutritional requirements due to increased intestinal losses, reduced intake, and malabsorption [21], suggesting that nutrient adequacy data should be interpreted cautiously. Further research is needed to define the appropriate intake levels of micronutrients like folate and vitamin B12 in patients with IBD. While the latest guidelines include specific protein and energy intake recommendations, there are no established values for other nutrients, leaving a gap in the nutritional recommendations for this population [21, 36].

Our results highlight an important finding: the association between vitamin B12 and lower ESR, a marker of inflammation, as well as with the absence of EIMs. This suggests that adequate B12 may have a protective role against inflammation [37]. Since ESR commonly rises in inflammatory conditions, the association observed could reflect the role of vitamin B12 in reducing Hcy [38, 39]. Additionally, the positive association between the absence of EIMs and the highest tertile (T3) of vitamin B12 intake also indicates the potential of vitamin B12 in attenuating Hcy, which is associated with autoimmune cascades [40]. Studies have shown that elevated Hcy levels in IBD result from pharmacological, dietary, and lifestyle factors, associated with low levels of vitamin B12 and folate [39, 41]. Moreover, research indicates that hyperhomocysteinemia exacerbates symptoms and inflammation in patients with rheumatoid arthritis, a frequent EIM in patients with IBD [42, 43].

Similarly, our study showed that folate was associated with the consumption of nutrient-rich foods, such as meats and fruits, and with iron and riboflavin consumption, indicating that healthy dietary patterns can contribute to reducing the risk of deficiency. Furthermore, total folate showed a negative association with the absence of EIMs. This is noteworthy, as folate plays a central role in Hcy metabolism [41], yet it did not show clinical associations comparable to those observed for vitamin B12. Studies show that folate supplementation can reduce Hcy by 25%, compared with 7% for vitamin B12. A meta-analysis by Chen et al. associated folate intake, but not vitamin B12, with lower heart attack risk, highlighting the role of Hcy in cardiovascular pathology [44].

In our dietary intake analysis, we were unable to identify correlations consistent with previous literature. A possible explanation is that folate may have a greater capacity to reduce Hcy concentrations and be associated with clinical outcomes when supplemented. Dietary patterns and individual variability in nutrient absorption may also have contributed to the absence of associations. A review on Hcy metabolism reiterates the impact of folate supplementation (0.5–5 mg/day); however, it provides limited evidence regarding food-derived folate [45], which may explain why folate intake from diet did not show clinical effects in our study, unlike vitamin B12.

Our analysis of biochemical parameters demonstrated that patients in remission showed higher serum folate and non-HDL-C levels, whereas patients with active disease had higher rates of B12 deficiency. The active phase, especially in patients who have already undergone ileal resection, may predispose them to increase the risk of B12 malabsorption [10], which could explain a greater proportion of active patients in the category. Evidence also associates low B12 with higher levels of inflammatory markers, such as IL-6 and C-reactive protein [37, 46, 47] supporting the hypothesis that deficiency may contribute to systemic inflammation and potentially worsen disease activity.

Regarding folate, previous studies indicate that CD patients tend to have lower serum levels [48], influenced by inadequate intake, malabsorption, increased requirements, inflammation, and medication use [10, 49]. These factors may be intensified during active disease, when appetite and intake often decline [41].

Furthermore, the remission group had a significantly higher mean of non-HDL-C than the active group. This may reflect a nutritional transition in patients in remission, where overweight and obesity become more prevalent. Our group previously reported that 55% of patients in remission were overweight or obese [14]. Obesity is associated with altered lipid profiles and long-term cardiovascular risk [50], and shares mechanisms with IBD, such as chronic inflammation, mesenteric fat infiltration, and adipokine dysregulation, that may influence lipid metabolism [51]. Conversely, increased cardiovascular risk and lipid alterations have also been described in active disease, driven by inflammation and treatment-related effects [31]. Overall, our findings emphasize that active disease and advanced phenotypes (B2/B3) are associated with lower B12 levels, higher metabolic risk, and greater nutritional vulnerability, whereas patients in remission show higher folate and non-HDL-C levels, potentially influenced by nutritional transition and weight gain. Given the high prevalence of overweight and obesity among patients in remission, we also explored whether BMI was associated with the metabolic risk categories for Hcy used in this study; however, no significant associations were identified. These findings suggest that Hcy-related risk in this cohort is more closely associated with nutritional deficiencies and disease-related factors than with BMI.

The lower B12 values observed in the B2/B3 phenotype align with prior evidence showing that ileal inflammation, pre-stenotic dilatation, and resections >20 cm markedly impair B12 absorption [52, 53]. As the ileum is the primary absorption site, structural and inflammatory alterations in this region likely drive both the nutrient deficiency and the higher metabolic risk observed in these patients.

Most patients in our sample were in the metabolic deficiency risk range for Hcy (140–300 pmol/L), although no differences were observed between the groups. Increased Hcy may promote Th17 differentiation and enhance pro-inflammatory cytokine production, contributing to complications such as strictures and fistulas [54, 55]. Therefore, there is an association between nutritional insufficiency and cardiovascular risk, regardless of the stage of the disease or deficiency status.

Thus, the risk category of patients in the B2 + B3 group with lower serum levels of vitamin B12 may have a bidirectional causality. There may be some pathophysiological relationship between higher Hcy concentrations and a worse prognosis of the disease. In addition, complications characteristic of the stricturing or penetrating disease could increase the risk of B12 depletion due to involvement of the ileum, intestinal inflammatory activity, the need for intestinal resection, and loss of tissue function [10].

Although these findings are predictive rather than conclusive, they emphasize the importance of nutritional assessment as part of the clinical routine for these patients since elevated Hcy and micronutrient deficiencies may affect the course of the disease and overall health. Dietary strategies to improve micronutrient intake could play a crucial role in managing CD by addressing malabsorption deficiencies, reducing inflammation, and supporting metabolic health. Therefore, the study suggests that folate and B12 levels could serve as markers for clinical characteristics and be associated with metabolic risk in CD patients.

Despite these findings, some limitations of our study should be considered. Dietary assessment based on a single 24-hour recall may not fully capture habitual intake [56], although single-day recalls have been used effectively in similar research [57, 58]. Additionally, the absence of serum Hcyone, one of our primary variables of interest, required the use of validated metabolic risk categories. While informative, these categories represent indirect estimates and cannot establish causal relationships. Future studies incorporating direct biomarkers such as methylmalonic acid and Hcy would deepen understanding of micronutrient metabolism and its role in CD progression.

## Conclusions

Our results demonstrate that patients with CD, according to the categorization of metabolic risk patterns, have a significant risk for elevated Hcy levels, with detrimental effects on overall health. This risk was particularly evident about vitamin B12 in patients with active disease, as well as in those with stenosing and/or penetrating behavior. It is important to note the link between Hcy and cardiovascular risk; however, other factors such as diet, excess weight, and medications may also play a role, which could explain the increase in non-HDL-C in patients in remission.

This study reveals an important connection between advanced CD phenotypes and metabolic risks, shedding light on a novel aspect of disease-related complications. Patients with B2 and B3 phenotypes may benefit from early screening and management of metabolic deficiencies to address elevated Hcy levels and reduce associated cardiovascular risks. Further research should explore therapeutic approaches to optimize metabolic health in these high-risk subgroups of patients, contributing to improving treatment strategies and helping reduce systemic complications in advanced CD.

Longitudinal studies are needed to establish causal relationships, explore how dietary changes could reduce these risks, improve patients’ quality of life, and incorporate stratified risk models for identifying patients more susceptible to vitamin B12 and folate deficiencies. These models could guide targeted interventions and optimize nutritional care.

## Data Availability

The data that support the findings of this study are available on request from the corresponding author.
